# Congruence Between County Dental Health Provider Shortage Area Designations and the Social Vulnerability Index

**DOI:** 10.5888/pcd21.230315

**Published:** 2024-06-27

**Authors:** Gabriel A. Benavidez, Elizabeth Crouch, Joni Nelson, Amy Martin

**Affiliations:** 1 Department of Public Health, Baylor University, Waco, Texas; 2 Department of Health Services Policy and Management, University of South Carolina, Columbia, South Carolina; 3 Department of Stomatology, Medical University of South Carolina, Charleston, South Carolina

**Figure:**
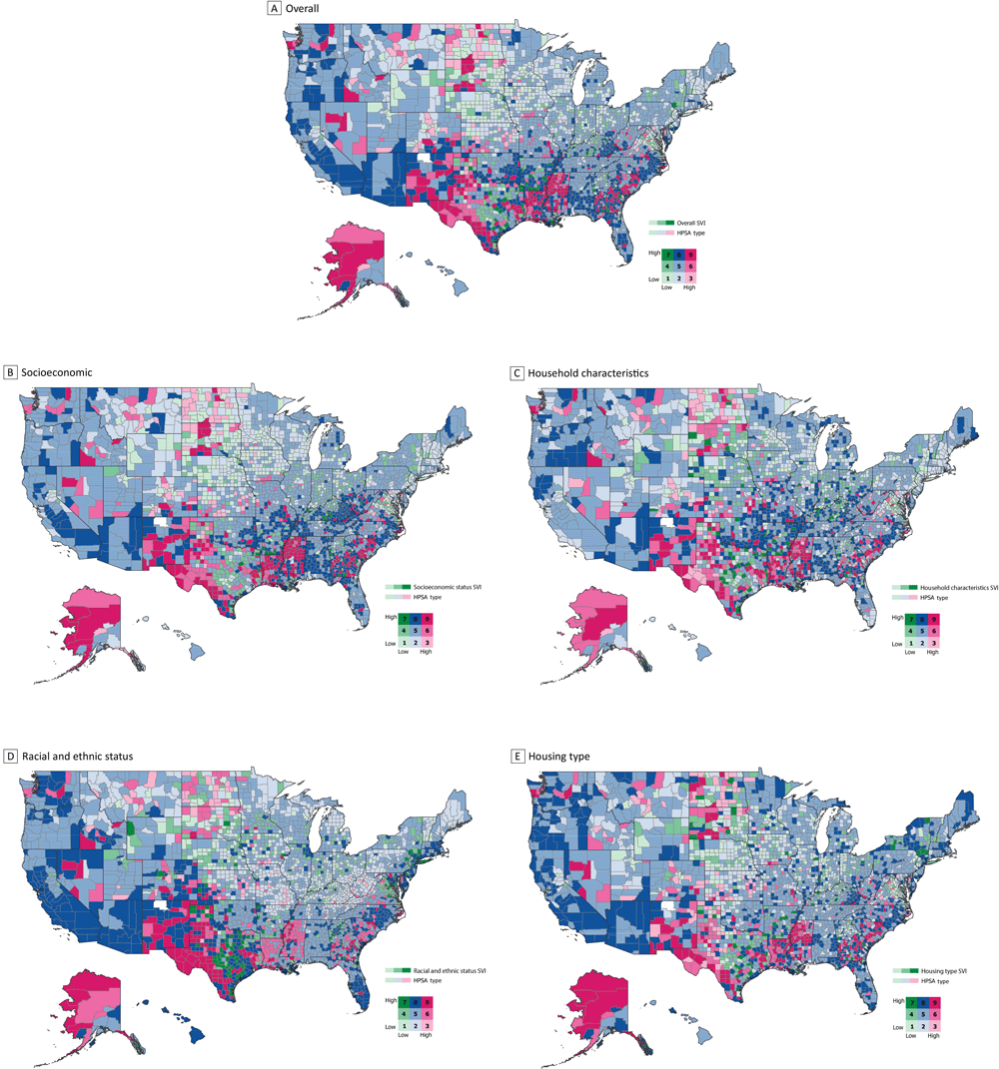
Bivariate maps of the United States show the relationship between Health Provider Shortage Area (HPSA) designations, extracted from the 2021–2022 Area Health Resource Files, and the Centers for Disease Control and Prevention’s 2020 Social Vulnerability Index (SVI) designations, completed in ArcGIS Pro (Esri). Legend values represent the following: 1 = no HPSA, low SVI; 2 = partial HPSA, low SVI; 3 = whole HPSA, low SVI; 4 = no HPSA, moderate SVI; 5 = partial HPSA, moderate SVI ;6 = whole HPSA, moderate SVI; 7 = no HPSA, high SVI; 8 = partial HPSA, high SVI; 9 = whole HPSA, high SVI.

## Background

Health Provider Shortage Area (HPSA) designations are given to regions or communities by the US Department of Health and Human Services to identify areas that have a shortage of health care professionals, including medical doctors, dentists, and mental health providers (1). These areas lack enough health care providers to meet the medical needs of their populations adequately (1). With this designation, these areas qualify for increased federal funding to support efforts to recruit and retain health care providers (1). A county may be designated as a whole-county HPSA if the entire county meets a minimum population-to-provider ratio with high need. It may be designated as a partial-county HPSA if populations or facilities with high needs exist at a subcounty level. Alternatively, it may be designated as a non-HPSA if there is deemed a low prevalence of high-need residents going without care.

Unfortunately, receiving these designations requires time-intensive documentation, so designations are updated infrequently, leading to inaccurate designations and misaligned incentives for health care providers (2). Additionally, HPSA designation criteria do not consider a wide range of social determinants of health (eg, uninsurance rates, employment, vehicle ownership), leaving the possibility that many vulnerable populations and communities are not receiving proper designations, resulting in a lack of resources to recruit and retain health care providers (3).

Oral health is an often overlooked yet vital component of comprehensive primary health care (4). Poor oral health disproportionately affects persons who have low income, are uninsured, are of a racial or ethnic minority, or reside in a rural area (4). Reasons for oral health disparities among these population groups are multifaceted but can be largely attributed to a lack of providers overall and a lack of providers who accept Medicaid (5). Accurate dental HPSA designations are crucial for allocating proper dental care resources to the communities that need them most. The purpose of our analysis is to examine the congruence between county-level dental HPSA designations and the Social Vulnerability Index (SVI) developed by the Centers for Disease Control and Prevention (CDC). The SVI is a well-studied and robust measure of a community’s social disadvantage that considers population measures of socioeconomic status, housing infrastructure, and racial demographics.

## Data and Methods

Our primary data sources for this analysis were the 2021–2022 Area Health Resource Files (AHRF) (6) and the 2020 county-level SVI (7). From the AHRF, counties were classified into 1 of 3 mutually exclusive dental HPSA groups: no HPSA, partial HPSA, or whole HPSA. By using the county-level SVI metric, which ranges from 0 (least vulnerable) to 1 (most vulnerable), we created a 3-category variable that classified counties as having low vulnerability (SVI ≤0.25), moderate vulnerability (SVI >0.25 and ≤0.75), or high vulnerability (>0.75). This classification methodology is also used by CDC to designate county-level vulnerability status. These vulnerability categories were created for the overall SVI measure and each individual domain that makes up the SVI (ie, socioeconomic status, household characteristics, housing type, transportation, racial and ethnic minority status). Using ArcGIS Pro v2.8 (Esri), we created bivariate county-level US maps to visualize the relationship between HPSA designation status (no HPSA, partial HPSA, whole HPSA) and county social vulnerability (low vulnerability, moderate vulnerability, high vulnerability). The use of bivariate maps enables a wholistic examination of the relationship between the SVI and dental HPSA designations across different geographic regions. By mapping both variables on the same map, one can identify geographic patterns, trends, and correlations between the 2 variables. We then examined the median SVI overall and domain scores by county HPSA designation stratified by rurality (metropolitan versus nonmetropolitan) based on Urban Influence Codes (8).

## Main Findings

A total of 3,135 counties or county-equivalents were included in this analysis. Most counties are designated as partial HPSAs for both metropolitan (74%) and nonmetropolitan (66%) counties (Table). When examining each theme of SVI scores, median SVI scores were significantly different across HPSA designation categories (Table). For example, in nonmetropolitan counties, the median overall SVI score was 0.24 for a no HPSA county, 0.54 for a partial HPSA county, and 0.76 for a whole HPSA county. This trend was repeated for each SVI theme across HPSA designations among the whole sample and when stratified by metropolitan status. To visualize the congruence between SVI and HPSA designation overall, we used a county-level bivariate map of the US. We identified 40 counties (map A, square 7) that had a high overall SVI (≥0.75) yet were classified as a no HPSA county — these counties were also predominantly in the southern United States. An additional 169 counties had moderate SVI with a no HPSA designation. Of the 480 whole HPSA counties, 47% (n = 224) had high SVI, 34% (n = 162) had moderate SVI, and 19% (n = 94) had low SVI. Similar trends were observed across other SVI domain variables (maps B–E, square 7) with only a small proportion of counties demonstrating high SVI scores while simultaneously being classified as a no HPSA county.

**Table:** Median County-Level Social Vulnerability Index (SVI) Score by Health Provider Shortage Area (HPSA) Designation and County Metropolitan Status^a^

Location/SVI Theme	Overall	No HPSA	Partial HPSA	Whole HPSA	*P* value^b^
**All counties (N = 3,135)^c^**					
Overall SVI	0.500	0.217	0.522	0.719	<.001
Socioeconomic status	0.500	0.218	0.524	0.751	<.001
Household characteristics	0.500	0.302	0.526	0.614	<.001
Racial and ethnic minority status	0.500	0.397	0.494	0.610	<.001
Housing type	0.500	0.250	0.539	0.545	<.001
**Metropolitan counties (n = 1,163)^d^**					
Overall SVI	0.441	0.204	0.509	0.584	<.001
Socioeconomic status	0.399	0.184	0.450	0.619	<.001
Household characteristics	0.353	0.227	0.382	0.462	<.001
Racial and ethnic minority status	0.612	0.504	0.651	0.536	<.001
Housing type	0.520	0.222	0.596	0.415	<.001
**Nonmetropolitan counties (n = 1,972)^e^**					
Overall SVI	0.537	0.237	0.537	0.762	<.001
Socioeconomic status	0.573	0.234	0.586	0.768	<.001
Household characteristics	0.579	0.377	0.599	0.648	<.001
Racial and ethnic minority status	0.429	0.348	0.385	0.626	<.001
Housing type	0.492	0.280	0.503	0.577	<.001

a Based on Urban Influence Codes (8).

b *P* value is based on Kruskal–Wallis test of rank sums for the overall SVI and each theme score.

c No HPSA n = 499; partial HPSA n = 2,156; whole HPSA n = 480.

d No HPSA n = 228; partial HPSA n = 855; whole HPSA n = 80.

e No HPSA n = 271; partial HPSA n = 1,301; whole HPSA n = 400.

## Action

While the methodology for defining HPSAs has been critiqued and is far from perfect, our analysis provides some evidence to suggest that current dental HPSA designations are appropriately placed. We find some evidence to suggest that a small proportion (<2.0%) of counties, mainly in the southern US, may be overlooked and may be missing out on much-needed resources to improve the availability of dental services in the area because of their no HPSA status while having a population of residents with high vulnerability indicators. Incorporating additional measures of the socioeconomic and built environment factors into HPSA designation consideration will likely only improve the current HPSA classification methodology. Future work should aim to examine the relationship between measures of oral health and HPSA designations to examine whether dental HPSA designations are not only located where they are intended but also where they are most needed.
